# Prevalence and clinical significance of proteinuria in intrahepatic cholestasis of pregnancy: A retrospective cohort study

**DOI:** 10.1097/MD.0000000000048544

**Published:** 2026-05-01

**Authors:** Resat Misirlioglu, Ali Cetin

**Affiliations:** a Department of Obstetrics and Gynecology, Haseki Training and Research Hospital, University of Health Sciences, Istanbul, Turkey.

**Keywords:** adverse pregnancy outcome, bile acids, intrahepatic cholestasis of pregnancy, preeclampsia, proteinuria

## Abstract

Intrahepatic cholestasis of pregnancy (IHCP) is the most common pregnancy-specific liver disease. This study aimed to determine the prevalence of proteinuria in patients with IHCP and to evaluate its association with adverse pregnancy outcomes. This retrospective cohort study included pregnant patients with gestational age > 24 weeks who were diagnosed with IHCP and completed 24-hour urine protein collection at Haseki Training and Research Hospital (January 2018–December 2024). Proteinuria was defined as ≥300 mg/24-h urine collection. Patients were categorized into 3 groups: non-proteinuric, isolated proteinuria (proteinuria ≥ 300 mg/24 h in the absence of hypertension), and preeclampsia (defined according to the American College of Obstetricians and Gynecologists [ACOG] criteria). The primary outcome was a composite adverse pregnancy outcome, including preterm delivery at <34 weeks, umbilical artery pH < 7.1, and emergency cesarean delivery due to fetal distress. Group comparisons were performed using the Kruskal–Wallis test for continuous variables and the chi-square or Fisher exact test for categorical variables. Multivariate logistic regression was performed adjusting for maternal age, twin gestation, and in vitro fertilization conception. Among 341 patients, 207 (60.7%) had no proteinuria, 105 (30.8%) had isolated proteinuria, and 29 (8.5%) had preeclampsia. Overall, 39.3% of IHCP patients demonstrated proteinuria. The distribution of IHCP severity, as classified by maximum total bile acid (TBA) concentration, was comparable among the 3 groups (*P* = .976). The demographic parameters were comparable between the groups. The preeclampsia group exhibited significantly higher composite adverse outcome rates (*P* < .001). Multivariate logistic regression demonstrated that preeclampsia was independently associated with composite adverse outcomes (adjusted odds ratio: 6.96, 95% confidence interval: 2.69–18.01; *P* < .001), whereas isolated proteinuria showed no significant association (adjusted odds ratio: 1.54, 95% confidence interval: 0.95–2.50; *P* = .079). Approximately 39% of IHCP patients exhibited proteinuria. Isolated proteinuria without hypertension was not an independent predictor of adverse pregnancy outcomes. In the absence of hypertension or other features of preeclampsia, the presence of proteinuria alone may not warrant escalation of care or accelerated delivery decisions in IHCP. However, prospective validation of these findings is needed before definitive clinical recommendations can be established.

## 1. Introduction

Intrahepatic cholestasis of pregnancy (IHCP) is the most common pregnancy-specific liver disorder, characterized by maternal pruritus and elevated serum bile acid levels.^[1,2]^ The disorder usually presents in the third trimester, with a global incidence of 0.1% to 15.6% and marked geographic and ethnic variability.^[3,4]^ The pathophysiology of IHCP is complex and involves interactions between genetic susceptibility, hormonal modulation, and environmental exposure, resulting in defective hepatic biliary transport and subsequent accumulation of bile acids in maternal circulation.^[5]^

The clinical ramifications of IHCP extend beyond maternal discomfort, as the condition has consistently been linked to adverse perinatal outcomes such as spontaneous preterm labor, meconium-stained amniotic fluid, fetal distress, and sudden intrauterine death.^[6,7]^ A landmark prospective population-based study by Geenes et al^[6]^ demonstrated that severe IHCP (serum bile acid concentration ≥ 40 μmol/L) was associated with significantly increased risks of preterm delivery (PTD; adjusted odds ratio: 5.39), neonatal unit admission (adjusted odds ratio: 2.68), and stillbirth (adjusted odds ratio: 2.58). Furthermore, accumulating evidence supports a dose–response relationship between maternal serum bile acid concentrations and fetal complications, informing contemporary clinical management protocols.^[7,8]^

Interest in the relationship between IHCP and preeclampsia has increased markedly in recent years. Multiple studies have demonstrated an increased incidence of preeclampsia in women with IHCP, suggesting a common pathophysiological mechanism.^[9-11]^ A recent systematic review and meta-analysis found that IHCP is significantly associated with preeclampsia (odds ratio [OR]: 2.64, *P* < .001).^[11]^ Both conditions share several common risk factors, biochemical abnormalities, and adverse pregnancy outcomes, which present diagnostic challenges in clinical practice.^[12]^ Diagnostic overlap is particularly problematic, considering that both entities may manifest with elevated hepatic transaminase levels, which requires careful differential diagnosis.

Proteinuria, considered a cardinal diagnostic criterion for preeclampsia, has traditionally been viewed as a pathognomonic feature that differentiates it from other pregnancy-related complications.^[13]^ Nevertheless, cholemic nephropathy provides a biological basis for kidney dysfunction under cholestatic conditions. Chronic cholestatic liver disease has been linked to tubulointerstitial nephropathies via mechanisms such as oxidative stress from bile acids, endothelial injury, and direct tubular toxicity.^[14-16]^ The discovery of bile acid receptors in renal tissue, including the farnesoid X receptor and G-protein-coupled bile acid receptor 1, further supports the possibility of bile acid-mediated renal effects.^[17]^

A recent study by Watad et al^[18]^ presented the first systematic assessment of proteinuria prevalence in IHCP and found that approximately 35% of patients had proteinuria; however, only some met the diagnostic criteria for preeclampsia. This finding challenges the traditional belief that proteinuria associated with elevated hepatic enzyme levels necessarily indicates preeclampsia, and raises important questions regarding the clinical significance of isolated proteinuria in IHCP.

Therefore, the main aims of the current study were to determine the prevalence of proteinuria in patients diagnosed with IHCP and to assess the association between proteinuria with and without concomitant hypertension and adverse pregnancy outcomes. We hypothesized that proteinuria in IHCP may represent a manifestation of cholemic nephropathy rather than occult preeclampsia and that isolated proteinuria in the absence of hypertension may not independently predict adverse outcomes.

## 2. Materials and methods

### 2.1. Study design and population

This retrospective cohort study was conducted at the Department of Obstetrics and Gynecology of the University of Health Sciences, Haseki Training and Research Hospital, Istanbul, Turkey between January 2018 and December 2024. The study protocol was approved by the Haseki Training and Research Hospital Scientific Research Ethics Committee (Decision No. 07-2025, dated January 30, 2025), and informed consent was waived owing to the retrospective nature of the investigation. The study was conducted in accordance with the Declaration of Helsinki and was reported in accordance with the STROBE (Strengthening the Reporting of Observational Studies in Epidemiology) guidelines.

Eligible subjects were pregnant women at a gestational age [GA] > 24 weeks who were diagnosed with IHCP and completed 24-hour urine protein collection during the index pregnancy. Cases were identified through systematic searches of electronic databases using the terms “intrahepatic cholestasis of pregnancy,” “cholestasis of pregnancy,” “elevated liver enzymes,” and “pruritus.” The diagnosis of IHCP was based on the occurrence of new-onset persistent pruritus associated with elevated total bile acid levels (TBA ≥ 10 μmol/L) in the absence of other known causes of liver disease (e.g., negative viral hepatitis serology [HBsAg, anti-HCV], normal hepatobiliary ultrasound, and exclusion of drug-induced liver injury). TBA measurements were obtained after an 8-hour fasting period, and the highest measured value during pregnancy was used for data analysis.

The exclusion criteria were higher-order multiple gestations (triplets or more), preexisting proteinuria in the prepregnancy period, primary hepatic disease unrelated to pregnancy, chronic kidney disease, and incomplete medical records. Singleton and twin pregnancies were included in this analysis.

### 2.2. Group classification

The participants were stratified into 3 groups based on proteinuria status and blood pressure measurements: IHCP without proteinuria (non-proteinuric group), defined as 24-hour urine protein < 300 mg; IHCP with isolated proteinuria (isolated proteinuria group), defined as proteinuria ≥ 300 mg/24 h in the absence of hypertension (systolic blood pressure < 140 mm Hg and diastolic blood pressure < 90 mm Hg) and without meeting the diagnostic criteria for preeclampsia; and IHCP with preeclampsia (preeclampsia group), retrospectively defined according to the American College of Obstetricians and Gynecologists (ACOG) Practice Bulletin No. 222 criteria^[13]^ as new-onset hypertension (systolic blood pressure ≥ 140 mm Hg and/or diastolic blood pressure ≥ 90 mm Hg on 2 or more occasions at least 4 hours apart after 20 weeks of gestation) accompanied by proteinuria (≥300 mg/24 h) and/or evidence of maternal end-organ dysfunction, including thrombocytopenia (platelet count < 1,00,000/μL), renal insufficiency (serum creatinine > 1.1 mg/dL or doubling of baseline), elevated liver transaminases (≥2 × upper limit of normal), pulmonary edema, or new-onset cerebral or visual disturbances. All medical records were reviewed retrospectively to verify that group classification was uniformly based on these objective criteria rather than on the treating physician’s subjective clinical impression alone.

Intrahepatic cholestasis of pregnancy severity was graded based on maximum TBA levels as mild (10–19 μmol/L), moderate (20–39 μmol/L), or severe (≥40 μmol/L), consistent with established classification methods.^[6,8]^

### 2.3. Technical protocol for clinical management

Hospitalization was required for all patients with suspected IHCP presenting at <37 weeks of gestation. The standardized evaluation protocol consisted of serial laboratory evaluations (complete blood count, coagulation profile, renal function tests, hepatic enzymes, and TBA) every 2 weeks, continuous fetal monitoring (cardiotocography) 3 times daily, ultrasonographic fetal growth evaluation every 1014 days, and 24-hour urine protein collection. Upon confirmation of IHCP, ursodeoxycholic acid therapy was initiated, according to institutional protocol.

Delivery timing was patient-specific and determined based on IHCP severity: 37 weeks if TBA was below 40 μmol/L, 36 weeks if TBA was 40 to 99 μmol/L, and 34 to 36 weeks for patients with very severe cholestasis (TBA ≥ 100 μmol/L).

### 2.4. Outcome measures

The primary outcome was a composite adverse pregnancy outcome consisting of any of the following: PTD before 34 weeks of gestation, umbilical artery pH < 7.1, or emergency cesarean delivery due to non-reassuring fetal status. The secondary outcomes were GA at delivery, birth weight, birth weight percentile, Apgar score < 7 at 5 minutes, neonatal intensive care unit (NICU) admission, meconium-stained amniotic fluid, fetal death, and neonatal death.

### 2.5. Statistical analysis

Continuous variables were assessed for normality using the Kolmogorov–Smirnov test and expressed as mean ± SD if normally distributed, or as median and interquartile range (IQR) if not. Categorical variables were presented as frequencies and percentages. Comparisons among the 3 groups were performed using the Kruskal–Wallis test for continuous variables and the chi-square or Fisher exact test for categorical variables, as appropriate.

Multivariate logistic regression was used to determine independent predictors of composite adverse outcomes, controlling for potential confounders including maternal age, twin gestation, and conception by in vitro fertilization (IVF). The non-proteinuric IHCP group served as the reference category for all logistic regression analyses. Results were presented as ORs and 95% confidence intervals (CI). Statistical significance was set at a 2-tailed *P*-value of <.05. All statistical analyses were performed using Python version 3.11 with SciPy and statsmodels libraries.

## 3. Results

### 3.1. Study population characteristics

During the study period, 341 patients met the inclusion criteria and were enrolled. The distribution among the groups was as follows: 207 patients (60.7%) in the non-proteinuric group, 105 (30.8%) in the isolated proteinuria group, and 29 (8.5%) in the preeclampsia group. Overall, 134 patients (39.3%) presented with proteinuria; of these, 105 (78.4%) had isolated proteinuria, without concomitant hypertension.

The baseline demographic and clinical characteristics are summarized in Table 1. Maternal age, prepregnancy body mass index, gravidity, parity, and previous IHCP or PTD history were comparable among the 3 cohorts. The rate of IVF conception was significantly higher in the preeclampsia group (*P* = .030; Table 1). Twin gestation showed a numerically higher proportion in the preeclampsia group, although the difference did not reach statistical significance (*P* = .232).

**Table 1:** Demographic and clinical characteristics of study groups.

Variable	Non-proteinuric (n = 207)	Isolated proteinuria (n = 105)	Preeclampsia (n = 29)	*P*-value
Age (yr)	28.6 ± 5.9	28.3 ± 6.0	28.0 ± 5.2	.684
BMI (kg/m^2^)	25.4 ± 4.0	25.3 ± 3.8	26.2 ± 3.5	.574
Gravidity	3 (2–4)	3 (2–4)	3 (1–3)	.312
IVF conception	8 (3.9%)	12 (11.4%)	3 (10.3%)	.030
Twin gestation	16 (7.7%)	11 (10.5%)	5 (17.2%)	.232
Previous IHCP	22 (10.6%)	14 (13.3%)	4 (13.8%)	.724
GDM	26 (12.6%)	10 (9.5%)	4 (13.8%)	.678

Data are presented as mean ± SD, median (IQR), or n (%).

BMI = body mass index, GDM = gestational diabetes mellitus, IHCP = intrahepatic cholestasis of pregnancy, IQR = interquartile range, IVF = in vitro fertilization, SD = standard deviation.

### 3.2. IHCP characteristics and laboratory findings

The IHCP-related parameters and laboratory findings are presented in Table 2. The GA at symptom onset and diagnosis did not differ among the groups. Importantly, the distribution of IHCP severity, as classified by the maximum TBA concentration, was comparable among the 3 cohorts (*P* = .976; Table 2). Median maximum TBA concentrations were similar across all groups (*P* = .890).

**Table 2:** Laboratory parameters by study group.

Variable	Non-proteinuric (n = 207)	Isolated proteinuria (n = 105)	Preeclampsia (n = 29)	*P*-value
TBA max (μmol/L)	22.9 (14.9–33.6)	22.0 (15.9–34.6)	22.4 (15.4–31.4)	.890
AST max (U/L)	79 (49–122)	89 (47–146)	86 (49–196)	.412
ALT max (U/L)	103 (62–169)	113 (65–169)	89 (46–159)	.356
Creatinine (mg/dL)	0.54 (0.46–0.62)	0.59 (0.49–0.68)	0.70 (0.62–0.77)	<.001
Urea (mg/dL)	21 (17–25)	25 (21–29)	30 (25–34)	<.001
24 h protein (mg/d)	161 (126–201)	520 (435–626)	810 (515–1166)	<.001

Data are presented as median (IQR).

ALT = alanine aminotransferase, AST = aspartate aminotransferase, IQR = interquartile range, TBA = total bile acids.

Hepatic transaminase levels were similar among the cohorts. However, renal function parameters demonstrated significant intergroup differences: serum creatinine and urea concentrations increased in a graded manner from the non-proteinuric to the isolated proteinuria and preeclampsia groups (*P* < .001 for both; Table 2). Twenty-four-hour urine protein excretion varied as expected among the 3 groups (*P* < .001).

### 3.3. Delivery and neonatal outcomes

Delivery and neonatal outcomes are presented in Table 3. Gestational age at delivery was significantly lower in the preeclampsia group compared with both the isolated proteinuria and non-proteinuric groups (*P* < .001). The rate of PTD before 34 weeks was correspondingly higher in the preeclampsia group (58.6%) than in the isolated proteinuria (20.0%) and non-proteinuric (12.1%) groups (*P* < .001). Emergency cesarean section due to fetal distress was numerically higher in the preeclampsia group, although the difference was not statistically significant (*P* = .298).

**Table 3:** Delivery and neonatal outcomes.

Variable	Non-proteinuric (n = 207)	Isolated proteinuria (n = 105)	Preeclampsia (n = 29)	*P*-value
GA at delivery (wk)	36.4 ± 1.6	36.0 ± 1.6	34.5 ± 1.2	<.001
PTD < 34 wk	25 (12.1%)	21 (20.0%)	17 (58.6%)	<.001
Emergency CS	34 (16.4%)	18 (17.1%)	8 (27.6%)	.298
Birth weight (g)	2602 (2228–2882)	2525 (2150–2781)	1825 (1642–2100)	<.001
NICU admission	31 (15.0%)	18 (17.1%)	10 (34.5%)	.034
Composite adverse outcome	75 (36.2%)	49 (46.7%)	23 (79.3%)	<.001

Data are presented as mean ± SD, median (IQR), or n (%).

CS = cesarean section, GA = gestational age, IQR = interquartile range, NICU = neonatal intensive care unit, PTD = preterm delivery, SD = standard deviation.

Neonatal outcomes reflected the differences in GA at delivery. The median birth weight was significantly lower in the preeclampsia group compared with the other 2 groups (*P* < .001). NICU admission rates were highest in the preeclampsia group (34.5% vs 17.1% and 15.0%; *P* = .034). No cases with Apgar scores <7 at 5 minutes were observed in any cohort. Two fetal deaths occurred in the non-proteinuric group, both in twin pregnancies with concomitant complications; no fetal or neonatal deaths occurred in the other groups.

### 3.4. Primary outcome analysis

Composite adverse pregnancy outcomes occurred in 147 of the 341 patients (43.1%). The rate was significantly higher in the preeclampsia group (79.3%) than in the isolated proteinuria (46.7%) and non-proteinuric (36.2%) groups (*P* < .001; Table 4).

**Table 4:** Multivariate logistic regression analysis for composite adverse outcome.

Variable	Crude OR (95% CI)	Adjusted OR (95% CI)	*P*-value
Non-proteinuric (reference)	1.00	1.00	–
Isolated proteinuria	1.54 (0.96–2.48)	1.54 (0.95–2.50)	.079
Preeclampsia	6.75 (2.77–16.45)	6.96 (2.69–18.01)	<.001
Maternal age (/yr)	–	0.95 (0.92–0.99)	.018
Twin gestation	–	0.73 (0.33–1.60)	.428

Adjusted for maternal age, twin gestation, and IVF conception. The non-proteinuric group served as the reference category.

CI = confidence interval, OR = odds ratio.

Multivariate logistic regression analysis, with the non-proteinuric group as the reference category and controlling for maternal age, twin gestation, and IVF conception, confirmed that preeclampsia remained independently associated with composite adverse outcomes (adjusted OR: 6.96, 95% CI: 2.69–18.01; *P* < .001). Isolated proteinuria showed no statistically significant independent association with adverse outcomes (adjusted OR: 1.54, 95% CI: 0.95–2.50; *P* = .079). Maternal age had a small but significant protective effect (adjusted OR: 0.95, 95% CI: 0.92–0.99; *P* = .018), whereas twin gestation showed no independent association with the composite outcome after adjustment (Table 4).

## 4. Discussion

This study provides novel insights into the prevalence and clinical significance of proteinuria in intrahepatic cholestasis of pregnancy (IHCP). In our cohort, 30.8% of patients with IHCP demonstrated isolated proteinuria (without concomitant hypertension), which is substantially higher than the reported prevalence of isolated gestational proteinuria in the general pregnant population (2–8%).^[19]^ When including patients with concomitant preeclampsia, the overall proteinuria rate was 39.3%. Importantly, 78.4% of proteinuric patients showed isolated proteinuria without concomitant hypertension, indicating that proteinuria could be an intrinsic clinical feature of IHCP rather than a feature of preeclampsia.

Our results are consistent with and expand upon those of Watad et al,^[18]^ who first reported an increased prevalence of proteinuria (34.5%) in patients with IHCP. The somewhat higher prevalence in our cohort (30.8% for isolated proteinuria; 39.3% overall) may reflect differences in the patient populations or diagnostic thresholds. Crucially, our study adds to the literature by demonstrating that isolated proteinuria, independent of preeclampsia, is not independently associated with adverse maternal-fetal outcomes. This insight has important implications for clinical management, as it suggests that proteinuria alone should not prompt escalation of care or premature delivery in patients with IHCP.

The biological plausibility of proteinuria as a manifestation of IHCP is supported by the concept of cholemic nephropathy. Elevated circulating bile acids may injure renal tubular cells through multiple mechanisms, including oxidative stress, direct cytotoxicity, and modulation of vasoactive mediator production.^[14,15,20]^ In an experimental model, Fickert et al^[14]^ demonstrated that urinary-excreted bile acids are the pivotal trigger for cholemic nephropathy in obstructive cholestasis, primarily damaging collecting duct epithelium through direct tubular toxicity and provoking progressive tubulointerstitial injury. The finding of increased serum creatinine and urea levels in a graded manner among the groups (non-proteinuric, isolated proteinuria, and preeclampsia) is consistent with a continuum of renal dysfunction severity, which may be partly attributable to these cholemic effects.

A notable finding of our study was the similar distribution of IHCP severity among proteinuria groups. The maximum bile acid concentration and proportion of patients with severe IHCP (total bile acids ≥ 40 μmol/L) did not differ significantly among the groups, suggesting that proteinuria development is not simply a consequence of more severe cholestasis. This dissociation between IHCP severity and proteinuria status supports the possibility that different pathophysiological processes underlie proteinuria.

Multivariate analysis clearly delineated the differential prognostic importance of proteinuria with and without hypertension. Preeclampsia showed nearly 7-fold higher odds of composite adverse outcomes, consistent with the well-established adverse effects of preeclampsia on both maternal and fetal health.^[13]^ In contrast, isolated proteinuria was only modestly associated with adverse outcomes, and this association was not statistically significant (*P* = .079). Although this *P*-value suggests a nonsignificant trend that warrants cautious interpretation, the adjusted OR of 1.54 (95% CI: 0.95–2.50) indicates that a clinically meaningful association cannot be definitively excluded with the present sample size. This pattern implies that the pathophysiological consequences of preeclampsia, rather than proteinuria per se, are responsible for the risk of adverse outcomes in IHCP pregnancies complicated by concomitant hypertensive disease.

The clinical implications of these findings are significant. First, the high rate of proteinuria in IHCP should alert clinicians that positive 24-hour urine protein collections may occur without necessarily indicating preeclampsia. Second, isolated proteinuria in the absence of hypertension should not automatically be considered a reason for changing management or early delivery in patients with IHCP, although close monitoring remains prudent. Third, meticulous differentiation of isolated proteinuria from true preeclampsia remains crucial, as only the latter is associated with substantially worse outcomes.

Our study has several strengths, including a relatively large sample size with comprehensive clinical characterization, standardized diagnostic criteria and management protocols, and systematic 24-hour urine protein collection in all subjects. Multivariate analysis controlling for possible confounders enhances the causal interpretation of the differential effects of proteinuria with and without hypertension.

This study has several limitations. The retrospective study design did not allow for the establishment of causality. The inclusion criterion requiring completion of 24-hour urine protein collection may introduce selection bias, as patients managed as inpatients or those with more severe presentations may have been more likely to complete this assessment, potentially limiting generalizability to outpatient IHCP populations. Furthermore, the retrospective application of diagnostic criteria for preeclampsia, although standardized using ACOG guidelines in the present analysis, is inherently limited by the completeness and accuracy of medical record documentation. The single-center nature may limit the generalizability to populations with different ethnic compositions or healthcare settings. The lack of angiogenic factor measurements (sFlt-1 and PlGF) prevents the biochemical distinction between preeclampsia and isolated proteinuria.^[21]^ Consequently, some patients classified as having isolated proteinuria based on blood pressure criteria alone may have had underlying angiogenic imbalance indicative of subclinical preeclampsia, which could not be identified without sFlt-1/PlGF ratio assessment. The incorporation of angiogenic biomarkers in future studies would enable more precise phenotypic classification. Additionally, proteinuria evaluation was not performed early in pregnancy; thus, it could not be determined whether the observed proteinuria represented de novo development. Future prospective studies with serial measurements of proteinuria and angiogenic biomarkers are required to overcome these limitations.

## 5. Conclusions

In conclusion, proteinuria is common in IHCP, occurring in approximately 39% of patients, and may represent a clinical feature of IHCP possibly due to cholemic nephropathy. Critically, isolated proteinuria in the absence of concomitant hypertension is not independently associated with adverse pregnancy outcomes, whereas preeclampsia is associated with a substantially increased risk of composite adverse maternal-fetal outcomes. These findings highlight the importance of careful clinical differentiation between isolated proteinuria and preeclampsia in the management of IHCP and suggest that, in the absence of hypertension or other features of preeclampsia, the presence of proteinuria alone may not warrant escalation of care or accelerated delivery decisions. However, prospective validation of these findings is warranted before definitive clinical recommendations can be established.

## Author contributions

**Conceptualization:** Resat Misirlioglu, Ali Cetin.

**Data curation:** Resat Misirlioglu.

**Formal analysis:** Resat Misirlioglu, Ali Cetin.

**Funding acquisition:** Resat Misirlioglu.

**Investigation:** Resat Misirlioglu.

**Methodology:** Resat Misirlioglu.

**Project administration:** Resat Misirlioglu.

**Resources:** Resat Misirlioglu.

**Software:** Resat Misirlioglu, Ali Cetin.

**Supervision:** Ali Cetin.

**Validation:** Resat Misirlioglu.

**Visualization:** Resat Misirlioglu.

**Writing – original draft:** Resat Misirlioglu.

**Writing – review & editing:** Resat Misirlioglu, Ali Cetin.
